# The Cynomolgus Macaque MHC Polymorphism in Experimental Medicine

**DOI:** 10.3390/cells8090978

**Published:** 2019-08-26

**Authors:** Takashi Shiina, Antoine Blancher

**Affiliations:** 1 Department of Molecular Life Sciences, Division of Basic Medical Science and Molecular Medicine, Tokai University School of Medicine, 143 Shimokasuya, Isehara, Kanagawa 259-1193, Japan; 2 Centre de Physiopathologie Toulouse-Purpan (CPTP), Université de Toulouse, Centre National de la Recherche Scientifique (CNRS), Institut National de la Santé et de la Recherche Médicale (Inserm), Université Paul Sabatier (UPS), 31000 Toulouse, France; 3 Laboratoire d’Immunologie, CHU de Toulouse, Institut Fédératif de Biologie, Hôpital Purpan, 330 Avenue de Grande Bretagne, TSA40031, CEDEX 9, 31059 Toulouse, France

**Keywords:** cynomolgus macaque, *Macaca fascicularis*, MHC polymorphism, experimental medicine, nonhuman primate models

## Abstract

Among the non-human primates used in experimental medicine, cynomolgus macaques (*Macaca fascicularis* hereafter referred to as *Mafa*) are increasingly selected for the ease with which they are maintained and bred in captivity. Macaques belong to Old World monkeys and are phylogenetically much closer to humans than rodents, which are still the most frequently used animal model. Our understanding of the *Mafa* genome has progressed rapidly in recent years and has greatly benefited from the latest technical advances in molecular genetics. Cynomolgus macaques are widespread in Southeast Asia and numerous studies have shown a distinct genetic differentiation of continental and island populations. The major histocompatibility complex of cynomolgus macaque (*Mafa* MHC) is organized in the same way as that of human, but it differs from the latter by its high degree of classical class I gene duplication. Human polymorphic MHC regions play a pivotal role in allograft transplantation and have been associated with more than 100 diseases and/or phenotypes. The *Mafa* MHC polymorphism similarly plays a crucial role in experimental allografts of organs and stem cells. Experimental results show that the *Mafa* MHC class I and II regions influence the ability to mount an immune response against infectious pathogens and vaccines. MHC also affects cynomolgus macaque reproduction and impacts on numerous biological parameters. This review describes the *Mafa* MHC polymorphism and the methods currently used to characterize it. We discuss some of the major areas of experimental medicine where an effect induced by MHC polymorphism has been demonstrated.

## 1. Introduction

Cynomolgus macaques (*Mafa*) have been increasingly used over the past decades due to the limited availability of Indian rhesus macaques following the export ban in 1978 [1]. After the ban, researchers tried to find alternative models by using rhesus monkeys from China as well as other species of macaques such as pig-tailed macaque and the cynomolgus macaque. Because of the extensive distribution of *Mafa* throughout South East Asia, animals of different origins were used in experimental medicine. Numerous companies, primarily in the Philippines, Vietnam, China, and Malaysia, specialized to breed cynomolgus macaques in captivity. An original population of *Mafa* can also be found on Mauritius. Macaques were introduced on this island around 400 years ago by Dutch and/or Portuguese sailors [2]. Recent molecular work confirmed that these animals, which were released in the wild, originated from either Java [3] or Sumatra [4]. The current population on Mauritius is derived from a small number—the estimate is around 20—founding animals [5,6]. The founding effect and total isolation of the Mauritius macaque produced a severe population bottleneck, resulting in a relatively poor genetic polymorphism when compared to the wild *Mafa* population of South East Asia [6]. The geographical distribution of cynomolgus macaques in South East Asia overlaps with that of rhesus monkeys north of the Kra isthmus. In this specific region, genetic studies demonstrated an introgression leading to gene flow from rhesus monkey males to *Mafa* [7,8,9,10].

Nonhuman primate models, including macaques, are frequently used in clinical and non-clinical testing because of their greater phylogenetic proximity to humans, when compared to mice or rats. The cynomolgus macaque is one of the nonhuman primate species used in clinical testing of organ allo-transplantations [11], stem cell allografts [12,13], the transfer of genes into stem cells [14], innovative vaccines [15] and immunotherapies [16], experimental infectious diseases [17], degenerative diseases and aging [18]. In all these fields, the use of a nonhuman primate model is justified by the absence of alternative animal models such as mouse or rat. Cynomolgus macaques are also used in non-clinical testing such as safety testing (toxicity, dependency, and reproductive and developmental toxicity testing), pharmacokinetic testing, and pharmacological efficacy testing. The proximity of the macaque and human immune systems has been a significant advantage in terms of the pertinence to extrapolate to humans the results obtained in macaques. For example, monoclonal antibodies for human therapy frequently cross-react with the macaque equivalent of the targeted human antigen [16]. However, decades of experimental data also demonstrate that specific aspects of the macaque immune system differ significantly from that of human. The example of the CD28 superagonist monoclonal antibody (TGN1412) is a paradigm of the dangers of extrapolating observations made in the macaque model to humans [19]. Despite the fact that this review focuses on the description of the *Mafa* MHC and highlights differences with its human counterpart, it is important to note that macaque immune related genes also differ from their human equivalents in many other loci, such as killer cell immunoglobulin-like receptors (KIRs) and leukocyte immunoglobulin-like receptors (LILRs), which coevolved with the MHC genes [20].

A detailed understanding of *Mafa* MHC polymorphism is essential for numerous experimental protocols where inter-individual histocompatibility must be met, or which involve antigenic peptide presentation by MHC class I or class II proteins. Indeed, numerous human studies have shown that MHC polymorphism is pivotal in allo-rejection of cells or tissues, vaccine responses, the control of infectious diseases, the development of autoimmune diseases and immune regulation during pregnancy. In all these areas, experimental medicine using the *Mafa* model has to take into account the MHC genetic variability of the animals used in the protocols. For example, in most therapeutic trials, it is crucial that animals in the treated and control groups are matched as closely as possible for their MHC type. In other cases, when an innovative immunosuppressive regimen is being tested to block allogeneic rejection, it is crucial to use donor/recipient pairs which are as incompatible as possible. It has been shown that the DRB incompatibility of macaques is correlated with the results of mixed lymphocyte cultures and that DRB genotyping facilitates the choice of donor recipient pairs [21]. 

The main problem which compromises the interpretation of medical research results based on animal models such as the *Mafa*, is that the impact of genetic polymorphism is not always taken into account. In captive breeding programs, genetic control usually refers to planned crosses that prevent the progression of consanguinity and promote the maintenance of genetic diversity. However, in order to improve the power of animal experiments while keeping the number of individuals used as low as possible, it is necessary to select animals sharing a common geographical origin and to systematically select animals with the experimentally appropriate polymorphic alleles in loci known to influence immune-related responses. The MHC genotype plays a key role in the selection of animals in all fields of medical research involving immune responses. 

This review focusses on the macaque cynomolgus MHC and examines its genomic similarities and differences with human MHC regions, the history of techniques developed to study the MHC polymorphism of *Mafa* and their application to various fields such as the study of regenerative medicine and experimental infectious diseases. 

## 2. Brief Review of Experimental MHC Polymorphism Milestones

In the early 70s, Hans Balner and his collaborators started the study of histocompatibility antigens in rhesus monkeys and chimpanzees [22,23,24,25,26,27,28,29]. The costs of breeding animals in captivity and the reduction in the number of feral animals that could be sourced, limited the overall use of chimpanzee and rhesus monkey models. These difficulties motivated the search for alternatives. In the early 70s, cynomolgus macaques could be sourced from Malaysia, Indonesia, and the Philippines. This triggered a renewed research interest in this species and Carolyn Keever and Eugene Heise characterized the Class I and Class II allo-antigens from alloimmune sera by working on a group of 277 feral Malaysian and Indonesian monkeys and 250 colony bred offspring for family studies [30,31,32]. In total, these serological studies led to the definition of three loci called A, B and C, consisting of 14, 10 and 6 alleles, respectively. The fact that in family studies the mixed lymphocyte culture (MLC) reactivity and survival times of skin grafts depended on donor-recipient CyLA compatibility, lead to the hypothesis that the serologically defined alleles were MHC gene protein products.

**Figure 1:**
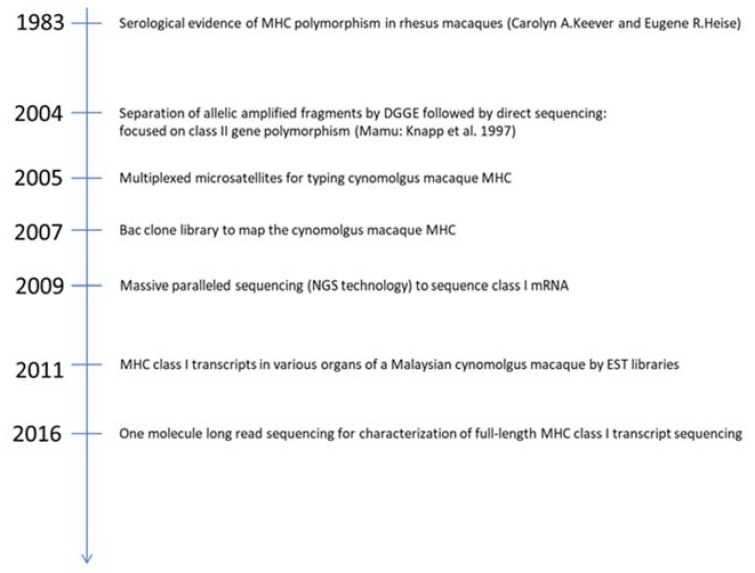
Milestones of cynomolgus macaque MHC studies.

Development of MHC genotyping started with the study of the DRB exon 2 polymorphism by DGGE (denaturing gradient gel electrophoresis) separating allelic amplified fragments, followed by direct Sanger sequencing [33], a technique first applied to the study of rhesus monkeys [34]. An alternative approach used microsatellites to characterize the polymorphic MHC region in cynomolgus from various geographical origins [35]. A BAC-based contig map of the *Mafa* MHC genomic region was published by Watanabe et al. (2007) [36]. Wiseman et al. published the use of massive parallel pyrosequencing of cDNA amplified fragments to genotype MHC of cynomolgus macaque as well as other species of macaques [37]. Alice Aarnink et al. characterized MHC class I transcripts of a Malaysian cynomolgus macaque from EST libraries [38]. Westbrook et al. reported on the use of full-length MHC class I transcripts using single-molecule real-time (SMRT) sequencing and described *Mafa* alleles in various populations [39]. The main steps in the MHC characterization of the cynomolgus macaque are presented in Figure 1.

## 3. Genomic Structure of the *Mafa* MHC Region and MHC Polymorphism

### 3.1. Length of the Mafa Region

**Figure 2:**
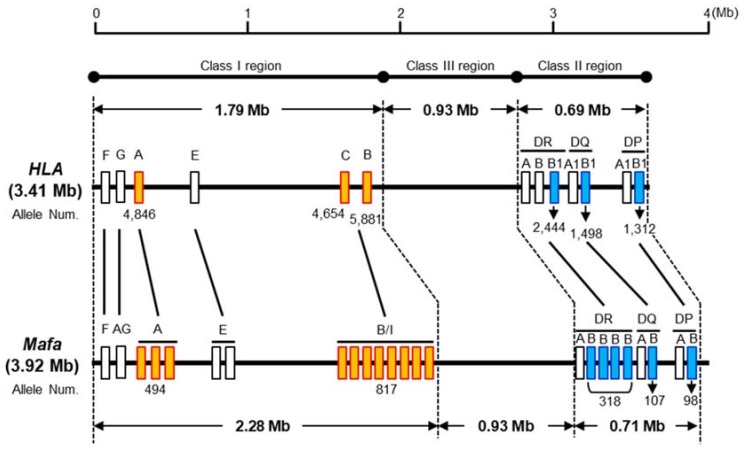
Comparative genome map of the HLA and *Mafa* regions. The number of *Mafa*-A, *Mafa*-B, and *Mafa*-DRB loci varies from one haplotype to another (*Mafa*-A from 1 to 3 loci, *Mafa*-B/I from 2 to 9, *Mafa*-DRB from 2 to 4). The numbers below the boxes indicate the number of alleles based on the IPD-IMGT/HLA version 3.35 and IPD-MHC version 3.2.0.0 database. The numbers of *Mafa*-A, *Mafa*-B and *Mafa*-DRB alleles correspond to the allele numbers of all *Mafa*-A loci (*Mafa*-A1 to -A6 and *Mafa*-A8), all *Mafa*-B loci (*Mafa*-B, -B11L -B12, -B16, -B17, -B20 and -B21), as well as all *Mafa*-DRB loci (*Mafa*-DRB*W, -DRB1, -DRB3, -DRB4, -DRB5 and -DRB6). For more details on allele numbers, see Table 1.

Figure 2 shows a comparative MHC genomic map, based on the nucleotide length of each of the MHC class I, class II and class III regions in human (derived from genomic information of the current genome NCBI database (GRCh38.p2)) and *Mafa* taken from the previously mentioned Watanabe et al. (2007) study [36]. The nucleotide lengths of the whole *Mafa* and HLA regions from the class I region to the class II region are 3.92 Mb and 3.41 Mb, respectively. The *Mafa* and HLA class II and class III regions are well conserved and extend over a 0.93 Mb and 0.69 to 0.71 Mb region, respectively. In contrast, the *Mafa* and HLA class I regions extend over 2.28 Mb and 1.79 Mb nucleotide lengths, respectively, and the specific lengths of the MHC-A and MHC-B/I subregions which include redundant gene duplication events differ significantly between the two species.

### 3.2. MHC Genes

The names of all thirty-three MHC loci and allele groups (18 class I: *Mafa*-A1, A2, A3, A4, A5, A6, A8, AG, B, B11L, B16, B17, B20, B21, E, F, G and I, and 15 class II: *Mafa*-DMA, DMB, DOA, DOB, DPA1, DPB1, DQA1, DQB1, DRA, DRB, DRB1, DRB3, DRB4, DRB5 and DRB6) in the *Mafa* region have been adopted by the Comparative MHC Nomenclature Committee as established by the International Society for Animal Genetics (ISAG) who are affiliated to the International Union of Immunological Societies (IUIS)-Veterinary Immunology Committee (VIC). Seven of these loci or allele groups (B11L, B16, B17, B20, B21, G and DRB6) do not express any RNAs. 

The *Mafa* equivalents of the classical HLA-A and HLA-B loci and the non-classical HLA-E, HLA-F, and HLA-G loci are designated *Mafa*-A, *Mafa*-B, *Mafa*-E, *Mafa*-F and *Mafa*-G, respectively. *Mafa*-G is considered a pseudogene and the functions associated with HLA-G may have been taken over by *Mafa*-AG, which shares similar features to HLA-G which is expressed in human placenta. In addition, eight splice variants have been observed in *Mafa*-AG [40]. To date, no ortholog of the HLA-C locus has yet been identified in the cynomolgus monkey or any of the other kinds of Old-World monkeys. 

The MHC class II loci are well conserved between the *Mafa* and HLA class II regions, with the exception of the MHC-DRB, MHC-DQA2 and MHC-DQB2 loci that were generated by different evolutionary processes [41]. The gene order within the *Mafa* and HLA class II orthologous gene regions is essentially identical.

The MHC haplotypes of the Mauritian and Filipino populations, were characterized by nucleotide sequencing of the MHC genes and consisted of seven major *Mafa*-class I haplotypes and class II haplotypes in the Mauritian population [42,43,44] and 84 major *Mafa*-class I haplotypes and 16 *Mafa*-class I haplotypes in the Filipino population [45,46,47]. One to three *Mafa*-A loci, one to eight *Mafa*-B and one to three *Mafa*-DRB loci were located in the duplicated subregions. However, information on MHC haplotypes in populations other than Mauritius and the Philippines is still lacking. 

### 3.3. Genes of MHC Class I Polypeptide-Related Sequences (MIC)

Three protein coding *Mafa*-MICA, *Mafa*-MICB and *Mafa*-MICB/A genes have been identified on the class III side of the *Mafa* class I region [36]. The *Mafa*-MICA and *Mafa*-MICB are orthologs of the human MICA and MICB genes, respectively, but *Mafa*-MICB/A is a hybrid of MICA and MICB generated by a crossing-over event with one breakpoint in the intron 3 region [48]. The MIC genes are polymorphic like the human MICA and MICB genes.

### 3.4. Non-MHC Genes

One hundred and twenty-two protein-coding non-MHC loci were identified in the HLA gene region, between GABBR1 and KIFC1 (Supplementary Table S1). The corresponding *Mafa* region contains 117 loci with 95.9% identity to the HLA sequences based on a comparison of the human GRCh38.p12 (GCF_000001405.38) and Macaca_fascicularis_5.0 (GCF_000364345.1) assemblies. In contrast, five HLA loci (PSORS1C1, LY6G6F, HSPA1A or HSPA1B, C4A and BTNL2) were not detected in the *Mafa* MHC genomic region. 

### 3.5. Nomenclature of Mafa Class I and Class II Alleles

The *Mafa* class I and class II allele names were assigned by the IPD-MHC NHP database following classical rules [49]. An example of the allele nomenclature is *Mafa*-A1*001:01:01:01N where *Mafa*-A1 is the MHC allele of the *Mafa* and encoded by the A1 locus. The first field after the asterisk defines the lineage number, the second field after the first colon describes a non-synonymous substitution between two sequences, the third field after the second colon describes a synonymous substitution between two sequences, and the fourth field after the third colon describes a substitution in the non-coding region between two sequences. The final letter ‘N’ indicates an altered level of expression such as for a null allele in the example given above.

### 3.6. Characteristics of Mafa Class I and Class II Allele Numbers

Table 1 shows the latest HLA and *Mafa* published allele numbers dating from 30 May, 2019. A total of 22,140 different allele sequence variations are annotated for HLA, with over 1000 different alleles published for the classical HLA loci, HLA-A, B, C, DRB1, DQB1 and DPB1. The class II α chain loci, HLA-DRA, DQA1 and DPA1, in contrast, are less polymorphic than the β chain loci, while the non-classical HLA loci, HLA-E, F, G, DOA, DOB, DMA and DMB, are not very polymorphic at all. In the case of *Mafa* only a total of 2196 different MHC allele sequence variants have been published to date. The MHC allele numbers in the macaque is expected to increase further because many novel alleles are still regularly identified in Vietnamese and Cambodian populations. In the *Mafa*-A1, B and DRB subregions, which result from gene duplication events, more allelic variants are observed for *Mafa*-A1, B and DRB*W than the other class I loci. The non-classical MHC loci, *Mafa*-E, F, G, DOA, DOB, DMA and DMB, like the human equivalent region, are not very polymorphic at all. Unlike the HLA orthologs, *Mafa*-DRA, DQA1 and DPA1 have similar allele numbers as the β chain loci (Table 1).

## 4. Diversity of Cynomolgus Macaque Populations

The polymorphic MHC region has been studied in several cynomolgus macaque populations (Table 2). Differences among these populations were clearly highlighted by microsatellite studies [35]. Genetic population studies of four distinct populations confirmed that the Mauritian cynomolgus macaque (MCM) population has the most restricted MHC polymorphism [50]. The Philippine macaque population also showed a restricted polymorphism compared to the Indonesian or Vietnamese *Mafa* populations [50]. The study of 36 autosomal microsatellites (18 spread across the MHC region and 18 outside MHC) in a sample of 254 individuals from four populations (Vietnam, Java, the Philippines, and Mauritius) suggested that the class III region was subjected to selection in the Philippine population [51]. This potential selection event was deduced from the difference between the observed F(st) and that deduced from a neutral model using two methods based on contrasting demographic models. The two approaches showed a signal of positive selection in the MHC class III region. This departure from a neutral model in the class III region was much more significant than the one previously reported for the DRACA marker, which may have hitchhiked due to its proximity to the class III region [50].

### 4.1. Study of MHC Class I Alleles in Various Populations

Many investigators have examined the MHC class I region of *Mafa* from various origins [38,47,52,53,54,55,56,57,58,59,60,61,62,63], but only a limited number of studies have looked at *Mafa* from Chinese breeding facilities [39,64,65]. Because no feral *Mafa* are found in China, Chinese breeding facilities are based on animals sourced from a variety of geographical locations, most likely from Thailand, Laos, Vietnam, Cambodia, and Malaysia, but also possibly from Indonesia and the Philippines. Cynomolgus macaques from Chinese breeding facilities therefore are genetically heterogeneous, and this heterogeneity may be particularly biologically relevant in highly diverse genomic regions like the MHC.

The study of class I cDNA sequences in various populations confirmed the complexity of *Mafa* Class IA and class IB haplotypes. Indeed, as in other *Cercopithecidae*, *Mafa* MHC haplotypes are characterized by a number of classical class I genes which are much larger than the corresponding human HLA haplotypes, the latter only encompassing three classical class I genes (HLA-A, -B, -C). The study of class IA and class IB alleles present in various *Mafa* populations indicated that a limited number of alleles and haplotypes are shared between populations. These studies also revealed sharing of several class I alleles with *Macaca mulatta*, or *M. nemestrina* [49]. 

### 4.2. MHC Class II Allele Sharing

Comparative study of DRB exon 2 in various *Mafa* populations confirmed that Mauritius and the Philippines have a restricted DRB polymorphism when compared to Vietnamese or Indonesian macaques [45]. The Vietnamese and Indonesian populations have a high diversity of DRB alleles [66,67]. Extensive interspecies DRB allele sharing was demonstrated by comparison *with Macaca mulatta* and *Macaca nemestrina* [49,68]. 

About one third of the *Mafa*-DRB exon 2 sequences are identical to rhesus macaque orthologs and one half of the *Mafa*-DPB1, *Mafa*-DQA1, and *Mafa*-DQB1 alleles are identical to *Mamu* orthologs [68]. Allele sharing between *M. fascicularis*, *M. mulatta* and *M. nemestrina* was also observed for the MHC DRA gene [69]. Despite extensive allele sharing, rhesus and cynomolgus monkeys differ in terms of MHC class II haplotypes, suggesting that after speciation, recombination processes generated species-specific haplotypes [68].

## 5. Comparison of MHC Polymorphism among Populations

As mentioned above, the Mauritius *Mafa* MHC is not very polymorphic when compared to the other *Mafa* populations [43,52]. Table 3 compares MHC polymorphism among the other three populations (Vietnam, Indonesia, Philippines). 

Vietnamese and Indonesian populations are better suited for biomedical research and non-clinical testing, in particular for exploring the impact of genetic polymorphisms. In contrast, the Mauritian and Filipino populations are better suited to biomedical research addressing genetic homogeneity because these populations have fewer alleles than other production areas [45,54,69,74]. Detection and breeding of MHC homozygotes is necessary for the development of vaccines and immunosuppression protocols, and for evaluating the usefulness of organs and regenerated cells derived from induced pluripotent stem (iPS) cells and embryonic stem (ES) cells [79,80]. It is for this reason that, Mauritius and Filipino cynomolgus macaques are considered to be the most suitable populations for use in biomedical research [3,4,81].

Polymorphism analysis results are based on 30 animals per population. The numbers indicate the number of MHC alleles detected in each population. The table shows that the Filipino population is less polymorphic than any of the other populations for any of the MHC genes tested.

## 6. Identification of MHC Region Homozygotes

**Figure 3:**
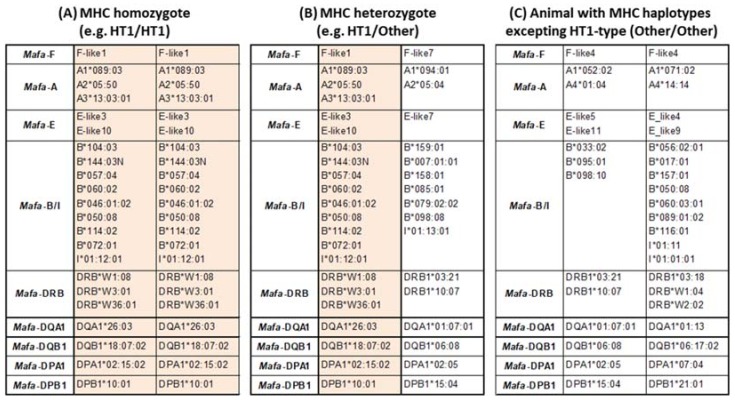
Examples of useful animals for non-clinical transplantation studies. Colored background shows MHC haplotype composition of HT1-type. (**A**) genotype of an HT1 homozygous animal, (**B**) genotype of an HT1 heterozygous animal (**C**) genotype of a heterozygous animal which has two haplotypes differing form HT1.

**Figure 4:**
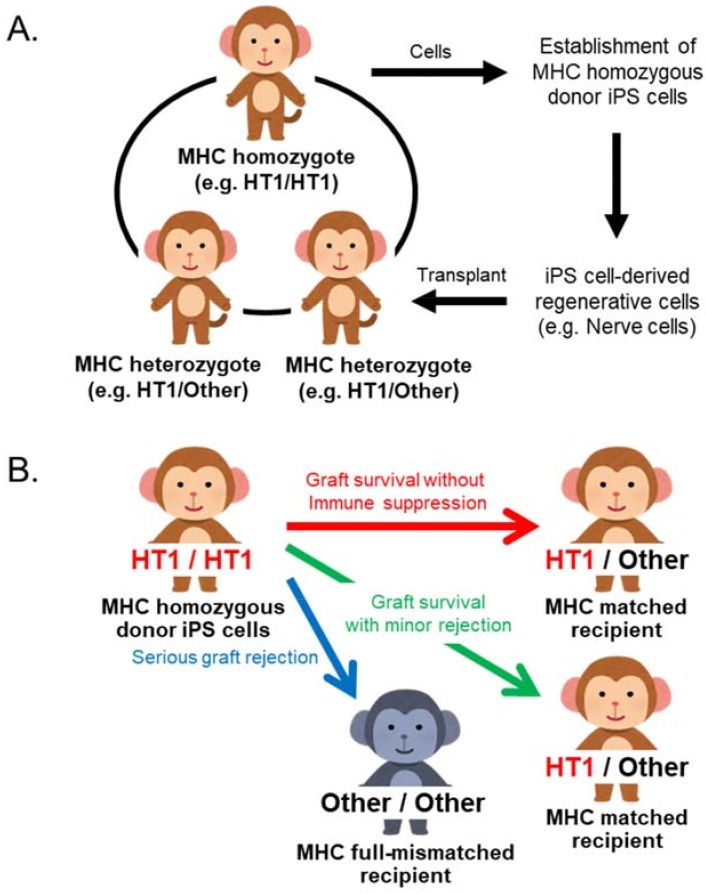
The iPS cell transplantation model and application for iPS cell transplantation study using MHC defined cynomolgus macaques. (**A**) This monkey model is used for analyses of engraftment rate of transplanted cells and inflammatory responses and used for evaluating safety and usefulness of iPS cell transplantation. (**B**) Summary of transplantation outcome between MHC matched recipient and mismatched recipient. MHC type mismatch in transplantation causes severe rejection. This transplantation model can be applied to the development of a therapy for suppressing graft rejection and an efficient protocol of an immunosuppressant.

MHC genotyping of animals originating from Mauritius using microsatellites was able to identify MHC homozygous animals [82,83]. In the case of Filipino animals, MHC genotyping based on high throughput sequencing of MHC class I and II cDNA (Ion PGM system Thermo Fisher Scientific) was used to identify MHC homozygous animals [47,84]. So far, a total of 207 MHC alleles have been identified from approximately 5,500 Filipino animals genotyped using this method, and we were able to detect 38 homozygous animals using any one of the 15 most frequently encountered MHC haplotypes (HT1 to HT15). Examples of Filipino animal MHC genotypes used for transplantation of induced pluripotent stem cells (iPS) are given in Figure 3 (see thereafter and Figure 4). 

## 7. Characterization of MHC Class I Expression Levels in Various Tissues and Conditions 

As detailed elsewhere in this review, the macaque MHC is characterized by its multiplicity of functional MHC class I genes. This is due to multiple rounds of duplications that resulted in the accumulation of classical class IA and class IB genes. Using high-throughput sequencing of exon 2 from amplified genomic DNA (190 bp), we characterized 23 classical class I genes from an MHC homozygous Mauritian animal (H2/H2) and 38 classical class I genes from a Malaysian cynomolgus monkey which was a putative MHC heterozygote [38]. Among these 38 classical MHC class I exon 2 variants only 25 had an intact open reading frame (ORF). Only 12 of these ORFs were associated with functional RNA sequences [38]. The availability of Expressed Sequence Tag (EST) libraries derived from mRNA extracted from six tissues of a Malaysian macaque (thymus, spleen, bone marrow, liver, heart and pancreas) [85], allowed the number of MHC class I sequences expressed in the various tissues to be estimated. MHC class I sequences deduced from EST libraries were compared to those obtained by massive pyrosequencing of MHC class I cDNA amplified from a lymph node RNA sample [38]. The study of the six EST libraries from various organs of the Malaysian animal studied revealed 16 MHC classical class I transcripts, 12 of which were associated with ORFs. The relative frequencies of classical class I transcripts in the lymphoid organs largely exceeded those observed in other non-lymphoid tissues. The thymus expressed the greatest number of transcript variants with relative frequencies of the various class I transcripts displaying a significant difference from those observed in the spleen (chi-square test, *p*  =  0.004). The relative frequencies of the MHC class I transcripts in the lymph node differed significantly from those observed in the spleen and in the thymus (chi-square test, *p*  <  0.0001 and *p*  =  0.002, respectively). No MHC classical class I gene transcript was detected from the EST library derived from pancreatic mRNA. The number of MHC class I functional transcripts defined in the Malaysian animal was within the estimated range determined using high-throughput sequencing, with between 17–23 of classical MHC class I genes transcribed in the MHC heterozygote cynomolgus macaques [37,52]. 

The evolutionary advantage of maintaining the efficient transcription of such a large variety of classical MHC class I genes is difficult to understand. From a classical point of view, the expression of MHC genes may have subtly adapted, to on the one hand avoid expressing an excessive variety of alleles leading to disproportionate levels of negative thymic selection and, on the other hand, an allelic diversity too weak to ensure the presentation of a large variety of exogenous peptides which is essential for mounting immune responses to the huge variety of microbial pathogens potentially encountered [86]. According to theoretical models, the ideal number of alleles seems to be around three MHC class I genes and three MHC class II genes [86]. One possible functional advantage to expressing such a variety of class I genes per haplotype is to allow the differential tissue expression of these genes. Differential MHC class I expression in distinct leukocyte subsets was studied by Green et al. (2011) using high-throughput pyrosequencing [87]. Transcription of certain MHC class I genes species varied significantly between different cynomolgus macaque leukocyte subsets. For example, the *Mafa*-B*134:02 RNA was virtually undetectable in CD4+ T cells, while it represented over 45% of class I transcripts in monocytes [87]. The authors also analyzed the expression of MHC proteins at the cell surface with fluorescent peptides capable of accessing the peptide grove of certain cynomolgus MHC class I proteins [87]. This demonstrated that distinct leucocyte subsets expressed MHC proteins differentially. A parallel study of human leukocytes revealed that expression of human HLA class I proteins does not significantly vary in the human cell subsets [87]. 

Another advantage of having a large number of class I genes per haplotype could be substantiated by the ability to induce expression of specific genes during particular infections. Changes in global gene expression were assessed for the brain, lungs, and spleen after aerosol exposure to the Venezuelan equine encephalitis viruses (VEEV) [88]. At day 3 post inoculation, the study revealed the induction of major histocompatibility complex (MHC) class I transcripts in the brain with no effect in the lungs or spleen [88]. Systematic study of the virus-dependent induction of MHC class I expression in cynomolgus macaque tissues remains to be explored. As it stands, we do not currently understand the overall functional advantages of expressing such a large variety of MHC class I genes per haplotype.

## 8. MHC and Experimental Infectious Diseases in Cynomolgus Macaque

Because the sets of peptides presented by MHC class I and class II proteins are allele dependent, the MHC polymorphism is associated with the control numerous infectious diseases. The influence that MHC polymorphism exhibits in controlling the SIV infection has been extensively studied and will be expanded on below. The peptide repertoire presented to macaque CD4+ T lymphocyte was also investigated in the case of tuberculosis [89]. Although this study, concluded that the immune repertoire of *Mafa* and of rhesus monkey largely overlap with that of humans, the association between immune responses to tuberculosis and the MHC class II polymorphism was not explored. Any future studies of innovative therapeutics or vaccines in the cynomolgus macaque have to take into account the genetic variability of *Mafa* MHC. Indeed, despite the phylogenetic proximity of macaques and humans, the fact that some macaques present a given set of peptides derived from a given infectious agent is not a guarantee that humans will present the same set of peptides. 

## 9. MHC and Control of SIV Infection

The years following the initial discovery of the AIDS epidemic in humans, were marked by reports of some individuals spontaneously controlling the infection over extensive periods of time. Multiple genetic association studies of the phenomenon demonstrated that the controller status is associated with specific HLA alleles, in particular the HLA-B57, HLA-B*58:01 and HLA-B27 alleles (for review see Martin and Carrington [90]). In contrast, the HLA-B35 allele is associated with poor control of viral infection and rapid progression to full-blown AIDS. The presence of protective HLA alleles was shown to be associated with a strong response of cytotoxic CD8+ T lymphocytes specific for particular HIV virus peptides, called dominant peptides. Multiple studies established that the HLA-B*57 B*58:01 and B*27 alleles present a set of peptides preferentially derived from gag protein, which become the dominant targets of cytotoxic CD8+ T cell responses. The proof of the effectiveness of the cytotoxic responses in controlling the infection comes from the demonstration of the progressive accumulation of viruses exhibiting non-synonymous mutations mapping to the regions encoding the dominant viral peptides presented by the protective HLA alleles, in controller patients. These mutated viruses circumvent the immune response because CD8+ T lymphocytes are now incapable of recognizing the mutated peptides since these can no longer be presented by the class I MHC molecules of the patient. These mutated viruses have a reduced replicative capacity (VRC viral replicative capacity) and the selection pressure exerted on the viruses by the controller’s immune response results in selecting viruses that accumulate compensatory mutations to help restore viral replication capacities. The gradual accumulation of these mutated viruses containing reversal mutations correlates with progression of the disease. Viruses encoding mutations which impart resistance to protective HLA alleles are transmissible and accumulate in populations affected by the epidemic so that the protective effect of HLA alleles decreases over time. This was first reported in Japan, where the protective role of allele HLA-B*51 disappeared with the rapid spread of mutated viruses [91]. In summary, in this arms race, the adaptive power of the virus far exceeds that of humans so that the population of viruses that prevails in a given infected human population gradually adapts to the HLA alleles associated with controlling the infection [91,92,93,94].

After the discovery of the SIV virus, the rhesus macaque of Indian origin became the animal model for virally induced AIDS. This animal model is by far the most frequently investigated and the best characterized non-human primate model. It reflects the observations in humans, with some rhesus monkeys also able to control the SIV infection for extended periods of time. Because the MHC polymorphism of rhesus monkeys was so well characterized, it was possible to demonstrate that in Indian rhesus monkeys the SIV controller phenotype was associated with the *Mamu*-A*01, *Mamu*-B*08, and *Mamu*-B*17 alleles. After SIVmac251/SIVmac239 challenge, rhesus macaques expressing these alleles presented with slow disease progression [95,96,97,98]. Interestingly, the *Mamu*-B*08 allele has very similar peptide presentation restrictions to those of the human HLA-B*27 allele [99]. As for the peptides derived from SIV, the peptides presented by the *Mamu*-B*08 allele include the homologs of the HIV peptides presented by the human allele HLA-B*27 [99,100]. In a manner, similar to that observed in humans, viruses carrying mutations mapping to the regions of the dominant peptides presented by *Mamu*-B*08, evade immune control when injected into *Mamu*-B*08 expressing animals [100]. Although viruses with multiple mutations have reduced replicative capacity than non-mutated viruses, they are nevertheless capable of inducing experimental AIDS in *Mamu*-B*08 animals [101].

Due to the increasing difficulty of accessing sufficient numbers of rhesus monkeys of Indian origin, researchers have attempted to use rhesus monkeys from China, but these animals differed too much from animals of Indian origin in terms of SIV-induced AIDS susceptibility. Since then, the cynomolgus monkey has started to be used more extensively as an alternative animal model for SIV infection, with the Mauritius cynomolgus macaque (MCM) being the most frequently used animal source. As previously mentioned, the MCM population experienced a sharp genetic bottleneck, as only a relatively small number of animals were initially introduced onto the island. With time, the total isolation of this macaque population favored a decrease in the total number of MHC alleles by genetic drift and/or local adaptations. Extensive investigation of MHC polymorphism in the current Mauritian macaque population has found that a mere seven of the ancestral MHC haplotypes have persisted. Due to recombination events accumulated over a period of 400 years on the island, a high percentage (about 31%) of recombinant haplotypes can be detected in this specific population [102]. The percentage of recombinant haplotypes allowed us to estimate the rate of recombination in the MCM population to be between 0.4 and 0.8% [102]. 

Many studies have reported an association between MHC polymorphism and control of the SIV infection in the MCM model. However, the conclusions drawn differ from one study to the other. Wiseman et al. 2007 demonstrated that MCM with identical MHC haplotypes mounted comparable cellular immune responses and maintained similar viral loads following infection with the widely used virus isolate SIVmac239 [75]. However, further studies found that MCM sharing MHC genotypes did not mount similar CD8 immune responses [103]. Moreover, M3 and M6 class IB and class II haplotypes were reported to be associated with resistance to chimeric SHIV89.6P challenge, while H2 and H5 class IB and class II haplotypes were associated with susceptibility to infection [104]. 

In an earlier study, Burwitz et al. demonstrated that the three most frequently found Mauritian MHC haplotypes share a pair of MHC class IA alleles, *Mafa-*A*25 and *Mafa-*A*29, which condition the presentation of SIV peptides to CD8+ lymphocytes [105]. They also reported on the accumulation of substitutions in the targeted peptides consistent with an immune evasion as a result of the selective pressure exerted by the cellular immune response [105]. Mee et al., showed that the MHC M6 haplotype in MCM is associated with a sustained control of the SIVmac251 infection [106]. Mee et al. subsequently demonstrated that the M3 haplotype was associated with a rapid control of SHIVsbg infection (significant differences in viral load apparent at 28 days p.i. despite comparable viral loads at day-14 p.i.) [107]. Aarnink et al. reported on the influence of the MHC genotype on the progression of experimental SIV infection (SIVmac239 strain), in the MCM [108]. The study of 44 animals allowed to detect an association between the plasma viral load at the set point and markers located in the MHC class IB region. Three MHC class IB haplotypes were significantly associated with lower (M2 and M6) or higher (M4) set point PVL values [108]. Two recent studies based on the same animal cohort highlighted evidence that several genetic factors outside the MHC are potentially involved in controlling SIV infections in the Mauritian macaque model [109,110]. In 2014, Alessandra Borsetti showed that MHC could influence the secretion of cytokines such as IL10 [111]. In the Mauritian macaque model, the MHC Class II M3 haplotype is associated with a lower acute and post-acute IL-10 level. Moreover, the class IA M3 haplotype is associated with a lower level of alpha defensins in the post-acute phase of infection [111]. The Mauritian MHC haplotypes associated with controlling the SIV infection vary from one study to the next. There are multiple causes to explain these discrepancies. Firstly, these studies are based on results from a limited number of animals that, in most cases, did not allow an analysis of all Mauritian MHC haplotypes. Secondly, the SIV strain used, the inoculation dose and the route of inoculation differ from one study to the other, so there are many confounding factors which likely bias results obtained in MHC association studies. Despite the discrepancies between the definition of the haplotypes associated with controlling SIV infection in the Mauritian macaque model, all published studies are in agreement with regards to the important role that the MHC class IB region plays in this mechanism. The host MHC class IB genetic background must be taken into consideration when designing and interpreting the results of any future SIV vaccine and therapeutic efficacy studies [112].

The beneficial effects of MHC heterozygosity in the control of experimental SIV infections were explored in the MCM model [113]. A statistically significant heterozygous advantage was demonstrated in HIV infected patients but it was difficult to explore the mechanism of this advantage because HIV strains are different for each individual HIV+ patient. In addition, individuals homozygous for a given HLA locus may also include different alleles at that locus and may also differ extensively from each other at other HLA loci. The advantage of the MCM model is that animals homozygous for the most frequent MHC haplotypes are not excessively rare. It is therefore possible to compare the evolution of the SIV infection in MCM homozygous for M1, M2 and M3 MHC haplotypes, with that in the heterozygous animals. Results from the numerous studies using this approach are nevertheless not so easy to synthesize because some conflicting observations were reported. In 2010, Shelby O’Connor et al. reported that MHC heterozygote MCM (five animals) have a lower plasma viral load than homozygous animals (eight animals) after experimental inoculation with the SIV239 strain [113]. In 2012, Budde et al. in a study on a larger number of MCMs (27 animals out of which 12 were homozygous) did not confirm the heterozygote advantage when all animals were considered together [42]. Restricting the comparison to M3/M3 (*N* = 6) and M1/M3 heterozygous animals (*N* = 6), showed that homozygous animals had a higher viral load at the set point than heterozygous animals [42]. Surprisingly, in 2012, Greene et al. reported that the T CD8+ lymphocytes from M1/M3 animals predominantly targeted SIV peptides restricted to the M1 haplotype [114]. This is not in favor of the theory arguing that the advantage of heterozygous individuals is imparted by the larger array of peptides presented and by heterozygotes who possess two different sets of MHC molecules instead of the one (as is case for homozygous animals). Although control of SIV infection differs between M1/M3 individuals and M3/M3 and M1/M1 individuals, it is not certain that the advantage of MHC heterozygosity results from an extension of the panel of viral peptides targeted by CD8 T lymphocytes. In 2016, the same group reported on immune “escaped” virus conditioned by MHC haplotypes [115]. They assessed the appearance of variants at the chronic phase of SIVmac239 infection in homozygous (M1/M1 or M3/M3) and heterozygous (M1/M3) MCM. The study demonstrated that a category of variants accumulated preferentially in M3/M3 individuals. By contrast, CD8+ lymphocytes specific for wild type (wt) peptides became more abundant in M1/M3 individuals. These results suggested that the accumulation of mutant escaped virus for a given peptide is associated with a progressive decrease of CD8+ lymphocytes specific for the wt peptide [115]. This association does not imply a cause and effect relationship between the two observations. Moreover, these observations are not consistent with the hypothesis of a more diversified CD8 response in heterozygous animals. Therefore, despite the possibilities offered by the MHC singularities of the MCM macaques, the detailed mechanisms of the heterozygous advantage in the fight against SIV infection remains elusive. 

In a more recent study, Li et al. reported that the protective effect of the M3 haplotype could be restricted to M3/M4 heterozygous animals [116]. However, this study was based on a very small number of animals (*n* = 12) among these were two animals showing the lowest plasma viral load at the set point which had an MHC M3/M4 genotype. Another recent study by Bruel et al. reported that long term control of SIV infection in MCM is not associated with an efficient SIV-specific CD8+ T lymphocyte response [117]. This contradicts a previous study reporting that a CD8+ specific response correlated with restriction of SIV replication in the MCM model [42]. 

## 10. Allogenic Lymphocyte Transfer 

The limited MHC diversity in the Mauritian macaque population, allows one to investigate adoptive transfers of allogenic lymphocytes between fully MHC matched animals (i.e., animals sharing two common MHC haplotypes). Indeed, the adoptive transfer of lymphocytes is indispensable for the study of the cellular immune response and is a promising strategy in particular clinical circumstance [118,119]. Initial experiments in MCM demonstrated that allogenic lymphocytes isolated from blood or various lymphoid organs did not subsist beyond 14 days in the full MHC-matched unrelated recipients [82,120]. Persistence of transferred lymphocytes was slightly prolonged in MHC-matched macaque siblings although it did not equal the persistence of autologous lymphocytes [83]. In the macaque model, only autologous adoptive transfer ensured the prolonged persistence of transferred lymphocytes in various organs [121]. However, neither allogenic nor autologous lymphocyte transfers of CD8+ T lymphocytes specific for SIV succeeded in controlling the SIV infection of the recipient. There are certainly multiple and complex reasons to explain this inefficacy. One can evoke the difficulty of transferred lymphocytes to localize to organs specifically affected by intense replication of SIV (i.e., the mucosae). Another difficulty in evaluating lymphocyte persistence in the recipient is that all experiments are based on labelling transferred cells with fluorescent dyes such as CFDA-SE (carboxyfluorescein diacetate succidimyl ester) or PKH67 (a compound characterized by a highly aliphatic alkyl tail attached to a fluorochrome). In both cases, labelling cells prior to transfer is susceptible to modify their cell surface properties as well as their metabolism. Alternatives to these fluorescent labels could be explored in order to avoid introducing CFDA-SE or PKH67 toxicity artefacts [122,123]. 

## 11. Role of MHC in Induced Pluripotent (iPS) Stem Cell Allografts

In Japan, an iPS cell stock project is underway to collect HLA haplotype homozygous iPS cells for treating HLA-matched patients [124]. The transplantation of differentiated cells from patient’s autologous iPS cells encompasses three major problems: it is expensive, it requires time-consuming processing for the preparation of differentiated cells and runs the risk of unmasking dormant inherited diseases. Ready-to-use HLA homozygous iPSCs are expected to solve these problems. In order to investigate the efficacy of ready-to-use MHC homozygous iPS cells, we established an iPS cell transplantation model system in cynomolgus macaque in which regenerative cells differentiated from iPS cells derived from MHC homozygotes are transplanted into MHC heterozygotes (Figure 4A).

This macaque transplantation system is used for transplantation of differentiated iPS cells such as retinal pigment epithelial cells [125,126], dopaminergic neuron cells [127,128], cardiomyocyte cell sheets [129] and cardiomyocytes [84]. Differentiated cells from MHC homozygous iPS cells were functional in vivo and minimal rejection was observed in MHC heterozygote recipients after transplantation in any of the cases. In contrast, transplantation of MHC mismatched animals often resulted in severe rejection (Figure 4B). In addition, the dose of immunosuppressant can be reduced in *Mafa* matched transplantations relative to *Mafa* mismatched transplantations [84,125,126,127,128,129,130]. This transplantation model can therefore be applied to the development of a therapy for suppressing graft rejection and is an efficient protocol for studying the effects of immunosuppressants in a nonclinical context.

We established a reproductive technique using intracytoplasmic sperm injection (ICSI) to maintain the necessary number of MHC-controlled cynomolgus macaques [131]. Namely, this is a technique for injecting MHC homozygous sperm cells into MHC heterozygous oocytes by microinjection. We have so far produced several MHC homozygotes and more than 10 MHC heterozygous animals using this technology.

## 12. Impact of MHC Polymorphism on Blood Counts

We investigated the impact of MHC polymorphism on various parameters obtained by complete blood count and flow cytometric analysis of lymphocyte populations in 200 unrelated cynomolgus macaques born in captivity from animals originating from the Philippines [132]. The MHC polymorphism was characterized using 14 microsatellites markers distributed across the MHC. The DRB locus was genotyped by denaturing gradient gel electrophoresis and sequencing. Among all cell count parameters analyzed, only two were associated with MHC polymorphism: CD4+ T lymphocytes and platelets [132,133]. The CD4+ T lymphocyte blood count was significantly associated with a DRACA-DRB haplotype (*p* < 8 × 10^−7^) [132], while the platelet count was significantly associated with two markers in the proximal class I region and class III region [133]. Examination of a Japanese cohort (14,967 subjects from the BioBank Japan Project) revealed several single nucleotide polymorphisms (SNPs) potentially associated with platelet count in a region homologous to the one we identified in the macaque model. It remains to be determined whether these associations are valid in other *Mafa* populations such as that of Mauritius. 

## 13. Macaque MHC and Experimental Autoimmune Diseases

The cynomolgus macaque is currently used as an animal model for several human autoimmune diseases [134,135,136]. However, until recently, only one study evoked a possible association between the development of the experimental disease and presentation of peptides by MHC proteins [137]. This preliminary study was based on only twelve animals originating from Mauritius (six class II M6 haplotypes and six without) [137]. After immunization with citrullinated peptides, the authors observed that the T-cell response was specifically directed against citrullinated peptides and not against arginine peptides. They reported that the presence of a valine in position 11 of the DRB alleles, and to a lesser extent of phenylalanine in position 13, led to an increased T-cell response to citrullinated peptides. It is important to note that the intensities of both the cellular (T cells) and humoral (production of antibodies) responses against citrullinated peptides were unrelated to the development of experimental arthritis in this model [138]. Indeed, a combined systemic and intra-articular immunization with citrullinated peptides is needed to induce experimental arthritis in the *Mafa* model. Cynomolgus macaques are also reported to develop collagen-induced arthritis, but any potential association between the development of this disease and MHC polymorphism remains to be investigated [136,139]. 

## 14. Impact of MHC on Cynomolgus Macaque Reproduction

In humans, several studies have suggested that fetuses that are MHC-compatible with their mothers may have a selective survival disadvantage compared to fetuses that inherit paternal MHC antigens which differ from maternal antigens (MHC semi-compatible pregnancies) [140,141,142]. The reproductive effects of feto-maternal histocompatibility (i.e., HLA sharing) is difficult to explore in humans due to the large number of HLA class I (HLA-A, HLA-B and HLA-C) and class II (HLA-DR, HLA-DQ and HLA-DP) alleles, requiring the study of many small subgroups and a loss of statistical power. Unlike the human population, the MCM population has a very limited MHC polymorphism (only seven founder haplotypes) as a consequence of the severe founder bottleneck experienced by the population. This limited polymorphism is of great value for exploring the impact of MHC compatibility on reproduction. We took advantage of this characteristic to explore the impact of feto-maternal MHC compatibility on reproduction in a captive *Mafa* colony of Mauritian descent [143].

We studied the MHC polymorphism in 42 macaque trios (male, female, and offspring) for whom the identity of the father was ascertained and with an equal theoretical probability of producing a totally compatible or semi-compatible offspring [144]. Animal MHC genotypes were deduced from the study of 17 microsatellite markers distributed across the MHC. Of the 42 offspring obtained, 11 were fully compatible and 31 were semi-compatible with their respective mothers. These proportions were clearly outside the 99% confidence interval when equiprobability is assumed for compatible and semi-compatible offspring. This departure from equiprobability suggests that *Mafa* offspring that are fully MHC-compatible have a selective survival disadvantage compared to offspring inheriting a paternal MHC haplotype distinct from the maternal haplotypes. By restricting the study of MHC feto-maternal compatibility to the MHC class II region, or the regions outside the latter, we found that departure from equiprobability of compatible/semi-compatible offspring only implicated the class I-III region [144]. The mechanisms at work in the negative selection of mother-compatible offspring in the MHC class I-III region remains to be identified. Due to the low density of microsatellite markers in the class I-III regions and the low frequency of recombinant haplotypes, the genomic region involved in the selection of offsprings has not been able to be mapped more precisely. The class I to III regions include numerous candidate genes. For example, in the Hutterite population, Ober et al. demonstrated that fetuses with identical HLA-B as their mothers were at increased risk of death in utero [145]. Similarly, to observations in humans, one could also evoke the impact of particular class I molecules, which can be expressed on the surface of trophoblastic macaque cells (a hypothetical homolog of human HLA-C). Genes in the MHC class III region (e.g., TNF alpha and TNF beta) play a role in reproduction [146,147] and could also explain our observations. The exact moment when such negative selection occurs (before or after fertilization or after implantation) remains an open question. The MCM represents an animal model that is accessible as well as phylogenetically close to humans, and amenable to experimental investigation of this issue.

## 15. Association of MHC and Drug Related Adverse Effects

Only one study has so far explored the association between delayed drug hypersensitivity and MHC in the *Mafa* model. The authors observed that nine out of 62 MCM exhibited clinical and histopathologic skin symptoms consistent with the drug-induced skin reactions observed in humans [148]. They studied the MHC genotype of the 62 animals and found an association between the drug induced skin reaction and the class IB region of Mauritian haplotype M3. The authors reported a high percentage of similarity (from 86% to 93%) between the class IB cDNA sequences associated with the Mauritian macaque M3 haplotype and the cDNA sequences of HLA-B alleles (HLA-B*57:01, B*15:02, B*58:01 and B*35:05) that had been previously reported to be associated with drug-induced hypersensitivity reactions in humans [149]. Obviously, a similarity at the cDNA level alone is not enough to draw conclusions about the mechanism of drug induced adverse reactions observed in the macaque model. A comparison of the amino acid sequences is also necessary. Moreover, the 62 macaques included in this study also received different drug treatments: either Metabotropic Glutamate Receptor 5 (mGluR5), Negative Allosteric Modulators or an unknown drug being tested by the authors [148]. Considering that only very few animals actually had adverse reactions to any of the drugs investigated, the study was unable to draw any significant findings when each of the drugs was considered separately. Notwithstanding these limitations, the Mauritian haplotype M3 could still be associated with a high risk of developing idiosyncratic drug-induced skin reactions.

## 16. Interactions between MHC Proteins and Various Receptors: Killer Cell Immunoglobulin-Like Receptors (KIRs) and Leukocyte Immunoglobulin-Like Receptors (LILRs)

As well as presenting peptides to the T cell receptor (TCR) of CD8+ T cells, classical MHC class I proteins also interact with KIRs and LILRs. KIRs are involved in the regulation of natural killer (NK) cell functions [150], while LILRs are involved in the regulation of antigen presentation by dendritic cells (DC) [151]. In the case of LILRs, it has also been shown that the strength of the human LILRB2 receptor and HLA-B protein interaction, contributes to control HIV-1 infections [151,152]. The strength of interaction is influenced by the HLA-B polymorphism, and strong LILRB2-HLA binding impairs the capacity of myeloid DCs to present antigens to T cells [152]. To our knowledge, only one study has to date explored the possible impact of LILR2 on the control of SIV infection in the MCM [153]. A longitudinal study of LILR2 expression after SIV inoculation revealed an up-regulation of LILRB2 and its MHC-I ligands on DCs in the early phase of SIV infection. The impact of this up-regulation on the capacity of DCs to present antigens to T cells remains to be explored [153]. The *Mafa* model offers the possibility of assessing the efficacy of anti-LILRB2 monoclonal antibodies aimed at suppressing this putative checkpoint in order to restore the full extent of DC’s capacity to present viral antigens to T cells.

As for KIRs, the human KIR locus encodes between 9 to 16 cell surface receptors expressed on NK and T cells, which interact with HLA molecules to transduce either inhibitory or activating signals. Inhibitory KIRs repress the NK cell-mediated killing of normal endogenous cells. In contrast, the downregulation of classical MHC class I protein expression at the surface of virally infected or malignant cells was shown to induce NK cell cytotoxicity, a process referred to as “missing self-recognition”. In this killing mechanism, NK cells detect, and subsequently destroy, aberrant cells that fail to express classical MHC class I molecules. Activating KIRs also interact with MHC class I molecules and may recognize cell surface alterations on unhealthy cells leading to their destruction by NK cells. Another well-characterized function of a specific type of activating human KIR receptor (KIR2DS1) is the activation of decidual NK cells through their interaction with HLA-C proteins [154,155,156]. This activation conditions the secretion of various cytokines (interferon gamma, vascular endothelial growth factor (VEGF) and others) by uterine NK cells. These cytokines are indispensable for the development of spiral arteries in the uterus as well as invasion of decidua by placental trophoblasts. One mystery which still requires resolution is the identification of the equivalent mechanism in macaques, as they do not have HLA-C gene orthologs. We have shown that *Mafa* reproduction is influenced by the MHC class I region [144]. It remains to be explored whether KIR-MHC interactions are also at work in the development of an efficient feto-maternal interface. 

The KIR and LILR receptors interact not only with conventional MHC-class I proteins but also with non-classical MHC class I proteins (HLA-E, F, G). The latter play a crucial role in the regulation of immune functions, and particularly in the activation of NK cells by their interaction with KIR and LILR receptors. The functional roles of these non-classical class I HLA proteins in the development of pregnancy, cancers, infectious diseases have recently been reviewed [157,158,159,160]. We have already indicated that the *Mafa*-E, *Mafa*-F genes, are found in all MHC macaque haplotypes and have low polymorphism, similarly to all other non-human primates. The functional role of *Mafa*-E (see below) has been particularly well established because of its involvement in infectious diseases and the success of innovative CMV (cytomegalovirus) vector-based vaccines, this is in contrast to the functional role of *Mafa*-F which remains to be investigated. As mentioned earlier in this review, there is no functional ortholog of HLA-G in macaques. However, the *Mafa*-AG genes, present in variable numbers in all *Mafa* MHC haplotypes, encode proteins that have tissue expression and functions similar to those of HLA-G.

Until recently, the complexity of the KIR locus in macaques has been detrimental to genetic association studies. Recent advances in molecular genetics now make it possible to genotype the macaque KIR locus. In the case of Mauritian macaque cynomolgus, a complete characterization of KIR haplotypes has been performed [20,161]. Although MHC/KIR epistatic interaction has been extensively studied in experimental SIV infection in the rhesus monkey model [162], it is still not understood in the cynomolgus macaque model. The rhesus monkey model has revealed a complex combined effect of MHC and KIR polymorphism in controlling SIV infections [162], and it is highly likely that the same epistatic interaction is at work for controlling SIV infections in the MCM model.

## 17. Roles of *Mafa*-E in Experimental Medicine

Similar to other non-classical MHC-Ib molecules, human HLA-E exhibits limited polymorphism (the two predominant HLA-E alleles differ by a single amino acid at position 107) [163]. HLA-E protein is known to present a 9-mer peptide derived from the signal sequences of HLA-A, B, C, and G proteins [164,165]. The HLA-E/signal peptide complexes are ligands of the CD94/NKG2 receptor which transduce an inhibitory signal to NK cells [166,167,168]. In addition to its essential function to protect healthy cells from NK cell mediated attack, HLA-E has also been demonstrated to present bacterial (*Mycobacterium tuberculosis* [169,170,171,172], *Salmonella enterica* [173]) and viral (EBV [174], CMV [175,176], and hepatitis C virus [177,178]) derived peptides to HLA-E restricted CD8+ T cells. The HLA-E–restricted CD8+ T cells recognize the pathogen-derived peptides by their TCR, secrete antiviral cytokines and kill infected cells [170,171,173,175,178], but the impact of these unconventional MHC-E– restricted CD8+ T cell responses on the control of infections remains elusive. 

Recently, the induction of SIV-specific MHC-E–restricted CD8+ T cell responses was demonstrated in the rhesus monkey after vaccination with an innovative anti-SIV vaccine based on a rhesus CMV (RhCMV) vector [179]. The *Mamu*-E–restricted CD8+ T cells response results in a robust control of SIV infection in approximately 50% of vaccinated Indian rhesus monkeys [180], confirming the role of *Mamu*-E in the presentation to CD8+ T cells of pathogen-derived peptides. More recently, MHC-E of Indian rhesus monkey, Mauritian cynomolgus monkey and human were compare for their expression at the surface of various cell types and their capacity to present virus-derived peptides to CD8+ T cells [181]. As is observed in the case of human CD4+ T cells infected with HIV, SIV infection of cynomolgus macaque CD4+ T cells induces an increase in MHC-E expression and the disappearance of classical MHC-I protein expression [181]. The macaque MHC-E molecules present identical SIV-derived peptides to MHC-E–restricted CD8+ T cells, leading to allogeneic and cross-species peptide recognition of MHC-E presented epitopes [181]. The functional similarity between human MHC-E and their macaque orthologs reinforces the argument supporting the relevance of macaque models (the rhesus monkey and the cynomolgus macaque) in the study of HLA-E in the fight against infectious pathogens [160].

In addition to its role as a non-classical MHC presenting protein, the MHC-E protein is also able to block NK cell activation by interacting with the inhibitory NKG2A receptor. Recently, a monoclonal antibody called monalizumab has been developed as a ckeck-point inhibitor that neutralizes the HLA-E/NKG2A interaction, thereby promoting NK cell activation against tumor cells and virally infected cells [182]. Despite the fact that the macaque model may be a suitable model for non-clinical trials, the cross reactivity of anti-human NKG2A with macaque NKG2A and NKG2C proteins will probably impede the use of this animal model in the future [183]. 

Another potential role of MHC-E peptide presentation to CD8+ effector T cells was reported in experimental allergic encephalomyelitis (EAE) in the marmoset model. In this model, EAE progression involves MOG-specific MHC-E restricted CD8+ CD56+ cytotoxic T cells activated by B cells infected with the EBV-related lymphocryptovirus virus CalHV3. The MOG (myelin oligodendrocyte glycoprotein) peptide (residues 40–48) is presented by the Caja-E molecules [184]. An in vitro study revealed that rhesus monkey MHC-E protein expression is enhanced by EBV infection of B cells [185]. Rhesus monkey EBV-infected B cells adopt a phenotype compatible with the presentation of the MOG 40–48 peptide [185]. In EBV free marmoset and macaque B cells, the MOG peptide is degraded by the endolysosomal protease cathepsin G and this destruction was interpreted as a tolerogenic mechanism. In contrast, in B cells infected with EBV-related lymphocryptoviruses, the induction of peptidyl arginine deaminase (PAD2 and PAD4) expression led to the citrullination of the MOG 35–51 peptide which protects the embedded MOG 40–48 peptide from the destruction by cathepsin G, leading in turn to the presentation of the citrullinated MOG 40–48 peptide by MHC-E [185,186]. Infection of B cells by EBV or EBV-related lymphocryptoviruses transforms these cells into APCs. This plays an essential role in the immunopathogenic process and is crucial to clarify our understanding of the therapeutic effects of anti-CD20 mAbs in multiple sclerosis [187]. The development of EAE models in the rhesus monkey and cynomolgus macaque would certainly help elucidate the role of EBV and MHC-E in the presentation of encephalitogenic MOG peptides in multiple sclerosis [186].

## 18. Conclusions

In conclusion, the study of *Mafa* MHC polymorphism has benefited from recent developments in molecular genetics. However, MHC gene duplication makes this region inaccessible to whole genome sequencing based on short reads. Techniques based on sequencing of very long fragments are required to reconstruct the complex cynomolgus MHC haplotypes. Moreover, many questions remain to be investigated, specifically in relation to the epistasis between MHC genes and other polymorphic loci such as KIR or LILR. In addition to the MHC, KIR and LILR loci, many other loci interfere with the immune response and their polymorphic variants need to be considered in the design of experimental medicine protocols [109,110,188]. The MHC polymorphism of macaque presents significant advantages for protocols aimed at establishing genetic associations. However, in most cases, experimental medicine in macaques aims at testing innovative treatments in this animal model. In that context, genetic variability of animals is a strong disadvantage and numerous efforts have been made to address this problem [131]. The recent developments in macaque cloning by somatic cell nuclear transfers will certainly play a determinant role in many protocols, for which the influence of the genetic background on the experimental results obtained needs to be limited [189].

## Figures and Tables

**Table 1:** Allele numbers of MHC genes in human and cynomolgus macaque.

Region	Allele Group	HLA Locus	Allele Num.	*Mafa* Locus	Allele Num.
Class I	MHC-F	HLA-F	44	*Mafa*-F	8
	MHC-G	HLA-G	68	*Mafa*-G	10
	MHC-AG	X	X	*Mafa*-AG	36
	MHC-A	HLA-A	5,018	*Mafa*-A1	320
				*Mafa*-A2	83
				*Mafa*-A3	30
				*Mafa*-A4	38
				*Mafa*-A5	8
				*Mafa*-A6	14
				*Mafa*-A8	1
	MHC-E	HLA-E	30	*Mafa*-E	16
	MHC-C	HLA-C	4,852	X	X
	MHC-B	HLA-B	6,096	*Mafa*-B	717
				*Mafa*-B11L	5
				*Mafa*-B16	11
				*Mafa*-B17	4
				*Mafa*-B20	2
				*Mafa*-B21	2
	MHC-I	X	X	*Mafa*-I	76
Class II	MHC-DR	HLA-DRA	7	*Mafa*-DRA	52
		HLA-DRB1	2,403	*Mafa*-DRB*W	160
		HLA-DRB3	217	*Mafa*-DRB1	82
		HLA-DRB4	108	*Mafa*-DRB3	13
		HLA-DRB5	77	*Mafa*-DRB4	10
				*Mafa*-DRB5	18
				*Mafa*-DRB6	35
	MHC-DQ	HLA-DQA1	149	*Mafa*-DQA1	100
		HLA-DQB1	1,560	*Mafa*-DQB1	107
	MHC-DO	HLA-DOA	12	*Mafa*-DOA	15
		HLA-DOB	13	*Mafa*-DOB	16
	MHC-DM	HLA-DMA	7	*Mafa*-DMA	11
		HLA-DMB	13	*Mafa*-DMB	7
	MHC-DP	HLA-DPA1	106	*Mafa*-DPA1	91
		HLA-DPB1	1,360	*Mafa*-DPB1	98
Total			22,140		2196

The HLA and *Mafa* MHC allele numbers refer to IPD-IMGT/HLA Release 3.36.0 and IPD-MHC Release 3.2.0.0 (2018-12-18) build 780, respectively.

**Table 2:** Cynomolgus macaque MHC polymorphism in various populations.

Reference	Year	Population of Macaques	Class I E	Class I F	Class I A	Class I B	Class I I	DRA	DRB Exon 2	DRB cDNA	Class II Others	Microsat.
Boyson et al. [70]	1995	Not specified	X									
Gaur and Nepom [71]	1996	Not specified							X			
Alvarez et al. [72]	1997	Not specified	X									
Otting et al. [73]	2002	Not specified									DQB	
Leuchte et al. [33]	2004	Mauritius							X			
Uda et al. [62]	2004	Not specified			X							
Krebs et al. [65]	2005	Vietnam, Mauritius, Chinese breeding facilities			X (RSCA) ^(1)^	X (RSCA)						
Uda et al. [61]	2005	Not specified				X	X					
Blancher et al. [21]	2006	Mauritius, The Philippines							X			
Sano K et al. [74]	2006	Indonesia, Vietnam, The Philippines									DPB1	
Doxiadis et al. [68]	2006	Indonesia Malaysia Thailand Java Sumatra Vietnam, Chinese breeding facilities							X		DPB1, DQA1, DQB1	
O’Connor et al. [43]	2007	Mauritius						X	X	X	DPA, DPB, DQA, DQB,	X
Wiseman et al. [75]	2007	Mauritius			X (RSCA)	X (RSCA)			X			X
Pendley et al. [59]	2008	Indonesia			X	X						X
Wiseman et al. [37]	2009	Mauritius			X	X						
Campbell et al. [53]	2009	The Philippines			X	X	X					
Kita et al. [54]	2009	Indonesia, Vietnam, The Philippines			X							
Aarnink et al. [69]	2010	Indonesia, Mauritius, The Philippines, Vietnam						X				X
Craeger et al. [66]	2011	Indonesia and Vietnam						X		X	DPA, DPB, DQA, DQB, cDNA	
Ling et al. [56]	2011	Vietnam									DOB, DPB1, DQB1	
Blancher et al. [45]	2012	The Philippines Java, Vietnam, Mauritius						X	X	X		
Ling et al. [76]	2012	Vietnam				X			X		DQB1	
Mitchell et al. [77]	2012	Indonesia			X (RSCA)	X (RSCA)						X
de Groot et al. [78]	2014	Indonesia, Indochina			X	X			X		DQA, DQB	X
Shiina et al. [47]	2015	The Philippines	X	X	X	X	X					
Karl et al. [64]	2017	Chinese breeding facilities			X	X	X					

RSCA: ^(1)^ Reference strand conformational analysis.

**Table 3:** Example of inter-population comparison based on MHC polymorphism.

Locus	Total Allele Num.	Vietnamese	Indonesian	Filipino
*Mafa*-A1	76	34	32	13
*Mafa*-DRA	22	17	_	12
*Mafa*-DRB	134	84	60	35
*Mafa*-DPB1	40	26	23	12

## Supplementary Materials

The following is available online, Table S1: Comparison of non-MHC loci in the MHC genomic region between human and cynomolgus macaque.
